# Collagen peptide promotes DSS-induced colitis by disturbing gut microbiota and regulation of macrophage polarization

**DOI:** 10.3389/fnut.2022.957391

**Published:** 2022-10-13

**Authors:** Xiaoxia Li, Luwen Cui, Guilin Feng, Shengnan Yu, Guanglong Shao, Ningning He, Shangyong Li

**Affiliations:** School of Basic Medicine, Qingdao Medical College, Qingdao University, Qingdao, China

**Keywords:** DSS-induced colitis, gut microbiota, macrophage polarization, collagen peptide, intestinal barrier

## Abstract

Ulcerative colitis (UC) is an inflammatory bowel disease caused by mucosal immune system disorder, which has increased steadily all over the world. Previous studies have shown that collagen peptide (CP) has various beneficial biological activities, it is not clear whether the effect of CP on UC is positive or negative. In this study, 2.5% dextran sulfate sodium (DSS) was used to establish acute colitis in mice. Our results suggested that CP supplementation (200, 400 mg/kg/day) promoted the progression of colitis, increased the expression of inflammatory factors and the infiltration of colonic lamina propria macrophages. Gut microbiota analysis showed the composition changed significantly and inflammation promoted bacteria was after CP treatment. Meanwhile, the effect of CP on macrophage polarization was further determined in Raw264.7 cell line. The results showed that CP treatment could increase the polarization of M1 macrophages and promote the expression of inflammatory factors. In conclusion, our results showed that CP treatment could disrupt the gut microbiota of host, promote macrophage activation and aggravate DSS-induced colitis. This may suggest that patients with intestinal inflammation should not take marine derived CP.

## Graphical Abstract

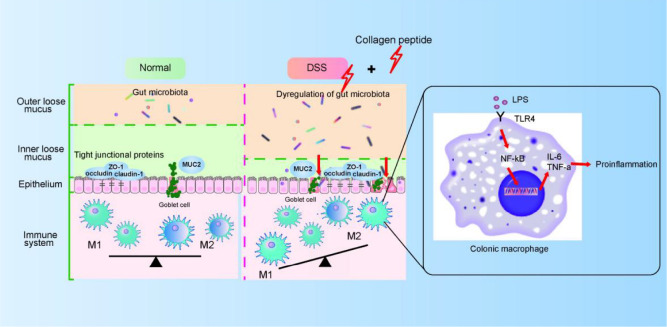

## Introduction

Inflammatory bowel diseases (IBD) are chronic inflammatory diseases of the gastrointestinal tract including ulcerative colitis (UC) as well as Crohn’s disease (CD) (1, 2). With the change of life style, the incidence rate of IBD is increasing which seriously affects the quality of life for patients (3). CD can be emitted in any part of the gastrointestinal tract and UC mainly affects the colon and rectum. Symptoms of UC are usually diverse, such as diarrhea, abdominal colic, rectal pain and stool bleeding. In

addition, some studies have shown that UC is an independent risk factor for colorectal cancer (4). However, the pathogenesis and treatment of UC remains a challenge.

The gastrointestinal tract is considered to be an ecosystem composed of gastrointestinal epithelial cells, mucus layer, immune cells and gut microbiota. Previous evidence showed the relevance between dysregulation of the gut microbiota with the progression of UC (5, 6). Besides, several studies believed that UC was also related to immune imbalance and macrophages played important roles in the development of UC (7, 8). The complexity, importance and interactions between symbiotic bacteria and the host immune system in UC are attracting more attention in recent years. Present therapeutic options such as the infliximab (anti-tumor necrosis factor α (TNF-α) antibody), 5-aminosalicylic acids, and the corticosteroid prednisone were mainly used to inhibit the immune system and relieve symptoms of patients (9, 10). Macrophages are one crucial component of host immune systems, and they are the gatekeepers of intestinal immune homeostasis since they can discriminate between innocuous antigens and potential pathogens (11). M1 macrophage could induce barrier defect which contributed to chronic intestinal inflammation in UC while IL-10-secreting M2 macrophages have better therapeutic efficacy in resolving intestinal inflammation. Several strategies to alleviate UC are focused on the polarization of macrophages (12–14).

Our previous research reported the production of Collagen peptide (CP) by the enzymatic degradation of Walleye Pollock skin (15). The content of hydroxyproline and proline was measured to be 10.30% and 8.99%, respectively. The molecular weight of CP varied from 500 to 5,000. CP are shown to exhibit the effects of anti-obesity (15) and gut microbiota regulation in HFD-fed mice (16) and anti-inflammatory activity in osteoarthritis and other joint disorders (17–19). UC as a disease closely related to gut microbiota, inflammation, and immunity, could CP exert anti-inflammatory roles in UC and other intestinal inflammatory diseases.

Herein, we observed that CP could increase the inflammatory response induced by dextran sulfate sodium (DSS) and accelerate the progression of colitis. This effect is related to the change of gut microbiota, polarization of macrophages and the increase of proinflammatory cytokines. Our study will lay a research foundation for the follow-up dietary guidelines and treatment of patients with UC.

## Materials and methods

### Materials and supplies

The preparation protocol of CP referred to our previous method (15). Antibodies against zonula occludens-1 (ZO-1, A11417), Claudin-1 (A11530), Occludin (A2601) and β-actin (AC026) were purchased from ABclonal (Wuhan, China). Antibodies against mucin-2 (MUC2, GB111965) was purchased from Servicebio (Wuhan, China). DSS for colitis model was purchased from MP Biomedicals (molecular weight: 36–50 kDa, Santa Ana, CA, United States). iNOS (18985-1-AP) and CD206 (60143-1-lg) antibodies for immumohistochemical staining were from Proteintech (Wuhan, China). CD80 (16-10A1) antibody for FACS analysis was from Biolegend (San Diego, CA, United States). Polystyrene latex beads with 2 μm diameter were from Sigma Aldrich (Shanghai, China). Antibodies against P65 (8242T) and p-P65 (3033T) used for western blot were purchased from Cell Signaling Technology while GAPDH (60004-1-lg) was from Proteintech (Wuhan, China). Lipopolysaccharide (LPS, L4391) was from Sigma Aldrich (Shanghai, China).

### Animals experiment

6-week-old male C57BL/6 mice (18–20 g) were purchased from Jinan Pengyue Laboratory Animal Breeding Company (Jinan, China). After 1 week of adaptation, the mice were randomly divided into four groups (*n* = 7): (1) NC group: normal control group; (2) DSS group: DSS-induced colitis; (3) CP-L: DSS-induced colitis and treated with low dose (200 mg/kg/day) of CP; (4) CP-H: DSS-induced colitis and treated with high dose (400 mg/kg/day) of CP. To induce UC, mice were fed 2.5% DSS in daily water of normal diet for 1 week. The dose was determined according to previous studies (15). Adjust the concentration of CP, and give CP 200 and 400 mg/kg/day (in 0.2 ml) by gavage to mice for 1 week. The weight of mice was recorded every day, and the serum and tissue were taken after the mice were killed, as described in the previous work (20).

### Cell culture and treatment

Raw264.7 cell line were cultured with the 1640 medium (HyClone, Greiner, Netherlands) supplemented with 10% FBS (Gibco, Rockville, MD, United States), 100 μg/ml of penicillin, and streptomycin at 37°C with 5% CO2 in a cell incubator. 100 ng/ml LPS was added to cell culture medium for 24 h before CP treatment. A concentration gradient of CP (0.1 mg/ml, 0.5 mg/ml, and 5 mg/ml) was set to study their roles on unstimulated or LPS-treated macrophages. Cells were collected for further analysis after 24 h or 48 h administration by CP.

### Histology analysis

The collected colon tissues were fixed in 4% phosphate-buffered formaldehyde solution for 24 h and embedded in paraffin. The 5 μm sections were stained with hematoxylin and eosin (H&E) or stained in Alcian blue by Sevicebio Technology Co., Ltd., (Wuhan, China). Then, the section of tissues after staining were observed using a microscope in 40 × and 200 × magnification (Olympus).

### Immunohistochemistry analysis

Immunohistochemical staining for the detection of polarization of macrophages was performed on Ventana’s Bench Mark XT automatic immunohistochemical staining machine using the avidin-biotin immunoperoxidase method. Briefly, the paraffin-embedded colon tissue sections were subjected to antigen retrieval and then were blocked and labeled overnight at 4°C with iNOS or CD206 antibody (1:200). After incubation with horseradish peroxidase (HRP)-conjugated secondary antibodies (Maixin, Fuzhou, China). The reaction was visualized using a 3,3′-diaminobenzidine (DAB) chromogen (Maixin).

### Enzyme-linked immunosorbent assay (ELISA) analysis

The collected serum by centrifugation was used for ELISA analysis. The cell samples were collected and washed with PBS for 3 times. After ultrasonic fragmentation, the samples were centrifuged at 4°C for 10 min (1,500 × *g*) and the supernatant was taken for ELISA detection. The ELISA kits of TNF-α (#H052-1), IL-1β (#H002), IL-6 (#H007-1-1) and IL-10 (#H009-1) were purchased from Nanjing Jiancheng Bioengineering Institute (Nanjing, China). The levels of proinflammatory cytokines were measured according to the instruction of ELISA kit.

### Real-time PCR analysis

Cultured cells were lysed by Trizol (NCM Biotech, Suzhou, China) and the RNA was extracted according to the manufacturer’s instruction. One microgram of total RNA from each sample was reverse transcribed in a final volume of 20 μL by Hifair II 1st Strand cDNA Synthesis SuperMix for qPCR (Yeasen, Shanghai, China). The polymerase chain reaction (PCR) amplification was carried out using Hieff qPCR SYBR Green Master Mix (Yeasen, Shanghai, China). All quantitative Real-time PCR (qRT-PCR) results were carried out in three duplicate wells and normalized to GAPDH. The primer of the related gene list is listed in Supplementary Table 1.

### Flow cytometry analysis

Flow cytometry analysis was performed as previously described (21). Briefly, 1 × 10^5^ cells were incubated for 60 min at 4°C with an antibody specific for CD80. Cells were then washed twice and resuspended in 500 μl of PBS. For each antibody, IgG of the same isotype from the same species was used as the isotype control. The experiment was performed using CytoFLEX S (Beckman Coulter) or Accuri C6 plus (BD) and analyzed by Flowjo software. For phagocytosis function detection, 1 × 10^5^ cells were collected after incubation with fluorescent beads for 8-h. Mean fluorescence intensity was used to indicate the phagocytic capacity of macrophages.

### Western blot

The protein samples were extracted using a cold RIPA lysis buffer (Solarbio, Beijing, China). Nanodrop 2000 spectrophotometer (Thermo Fisher Scientific, Inc., Waltham, MA, United States) was used to determine the protein concentration for each sample. Western blotting analysis was performed as previously described (20). In brief, protein samples (30 μg) were loaded to SDS-PAGE and transferred to a PVDF membrane. The membrane was incubated using the primary antibodies and the secondary antibodies to incubate separately for further detection. Protein expression of bands were quantified by Image J software (NIH, Bethesda, MD, United States).

### Gut microbiota analysis

Before the end of the experiment, the fecal samples of mice were collected. The collected fecal samples were treated with liquid nitrogen and stored at −80°C. As previous methods described, The Stool DNA isolation kit was used to extract the DNA from the acquired fecal samples (Tiangen Biotech Co., Ltd., Beijing, China) and next-generation sequencing library preparations and Illumina MiSeq sequencing were conducted at Novomagic (Shenzhen, Guangzhou, China) (15). The QIIME program (v1.9) was used to analyze the sequence. The 97% identity was used as default to cluster the sequences into operational taxonomic units (OTUs). Principal coordinate analysis (PCoA) based on weighted UniFrac distance matrices (R version 2.15.3) showing beta diversity. Relative abundance and functional prediction (Tax4Fun) were calculated by the Novomagic cloud platform (Shenzhen, Guangzhou, China).

### Statistical analysis

All graphics in this study were formatted with Adobe Illustrator 2020 (San Jose, CA, United States). The contrast between multiple groups was used one-way ANOVA (post-test: Tukey) in GraphPad Prism (V5, La Jolla, CA, United States). A value of *P* < 0.05 was considered significantly different.

## Results

### Collagen peptide promote the progression of DSS-induced colitis in mice

**FIGURE 1:**
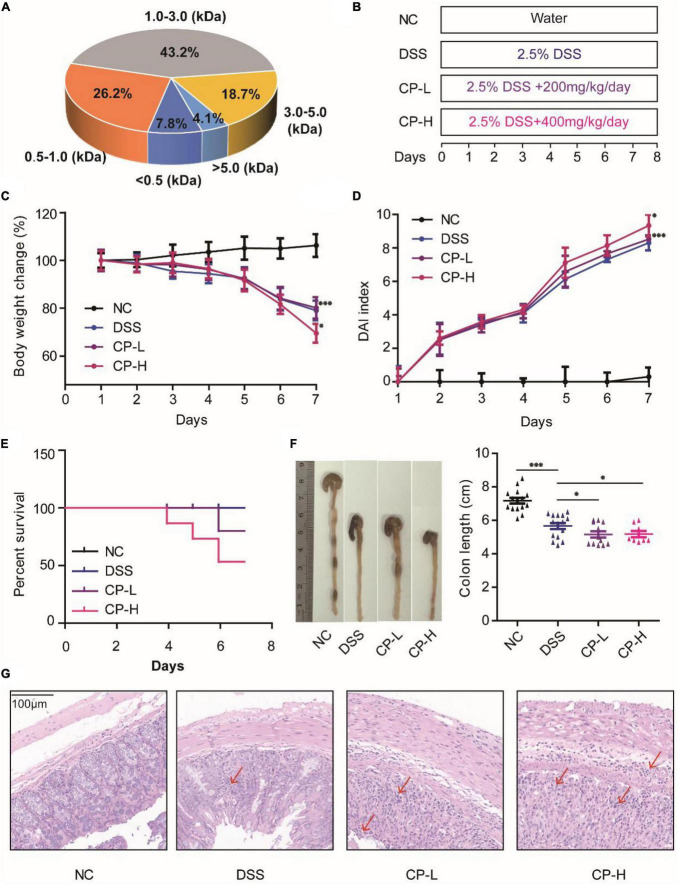
CP aggravated UC progression in DSS-induced colitis mice. **(A)** Molecular weight of CP. **(B)** Designed experiment flow. Body weight change **(C)** and DAI index **(D)** for 7 days DSS induction. **(E)** Survival rate of four experiment groups. **(F)** Colon pictures and colon length of four experiment groups. **(G)** Representative H&E staining. Compare with DSS group, *P < 0.05, ^**^P < 0.01, ^***^P < 0.001.

Collagen related products are the safe ingredients of drugs and foods recognized by the center for food safety and nutrition of the United States Food and Drug Administration (FDA). The CP used in this study are obtained from Walleye pollock skin and its molecular weight varies from 0.5 to 5 kDa (Figure 1A). To observe the effect of CP on colitis, DSS-induced acute colitis model was established in mice (Figure 1B). After DSS induction, the weight of mice decreased significantly (Figures 1C, *P* < 0.001), the situation of blood in stool was obvious (Figures 1D, *P* < 0.001), the survival ratio of mice decreased (Figures 1E, *P* < 0.05) and the length of colon shortened (Figures 1F, *P* < 0.001). After low-dose and high-dose CP treatment, the severity of DSS-induced colitis was significantly increased, the body weight was further reduced, the blood in stool and the mortality of mice were further increased, and the length of colon was further shortened. To study the effect of CP on the histology of colitis in mice, H&E staining was performed (Figure 1G). Compared with NC group, histopathological examination in DSS group showed inflammatory cell infiltration, glandular loss, mucosal epithelial necrosis, and loss. CP supplementation significantly increased severe histological injury, manifested by increased number of glands, destroyed integrity of mucosal epithelium and increased infiltration of inflammatory cells. Taken together, these results suggested that CP promotes the development of DSS-induced colitis.

### Collagen peptide did not aggravate the destruction of intestinal barrier in DSS-induced colitis mice

**FIGURE 2:**
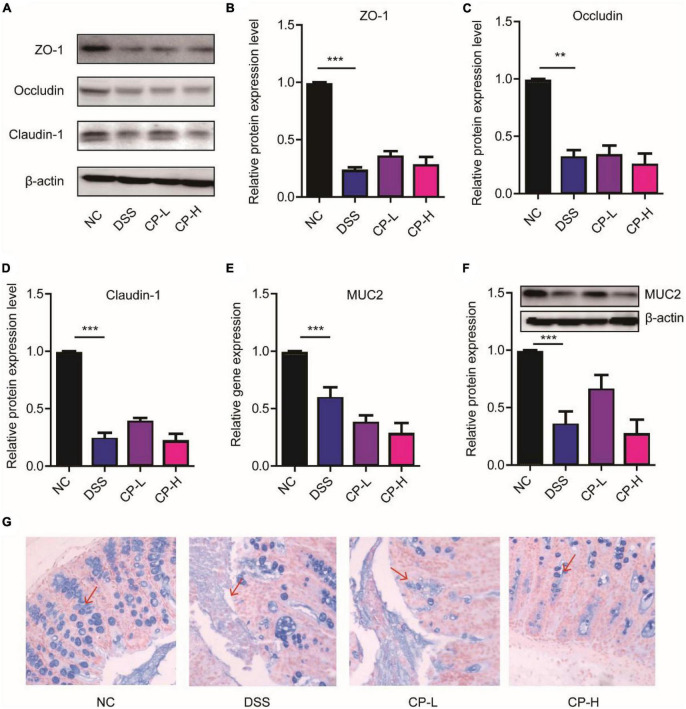
CP maintained the colonic epithelial barrier. **(A)** Western blot results of ZO-1, Occludin, Claudin-1 (*n* = 5). Quantitative analysis of western blot image for ZO-1 **(B)**, Occludin **(C)**, Claudin-1 **(D)**. **(E)** Real-time PCR results of MUC2 (*n* = 5). **(F)** Western blot and its quantitative analysis of MUC2 (*n* = 5). **(G)** Representative images of Alcian blue staining. Compare with DSS group, **P < 0.01, ***P < 0.001.

The destruction of colonic epithelial barrier function is an essential factor leading to the occurrence and aggravation of inflammation (22, 23). The tight junction (TJ) between colonic epithelial cells is an important structure to maintain intestinal barrier function. At the same time, mucus layer is also an important part of intestinal barrier function (24, 25). It is the first line of defense against symbiotic microorganisms and invasive pathogens. To study the effect of CP on intestinal barrier function, we observed the effect of CP on TJ protein expression and mucus layer thickness. DSS induction can significantly reduce the expression of TJ protein including ZO-1, Occludin and Claudin-1. TJ proteins were kept by both high-dose and low-dose CP administration in a state of low expression (Figures 2A–D). After DSS induction, the gene and protein expression of MUC2 in colonic mucus decreased significantly, and the state of low expression of MUC2 depended on CP treatment (Figures 2E,F). Meanwhile, the results of Alcian blue staining were consistent with the expression of MUC2 (Figure 2G). The results showed that CP treatment could maintain the decrease of TJ protein expression and mucus layer thickness caused by DSS, and then affect the stability of epithelial barrier structure and the change of epithelial permeability.

### Collagen peptide changed the composition of gut microbiota

**FIGURE 3:**
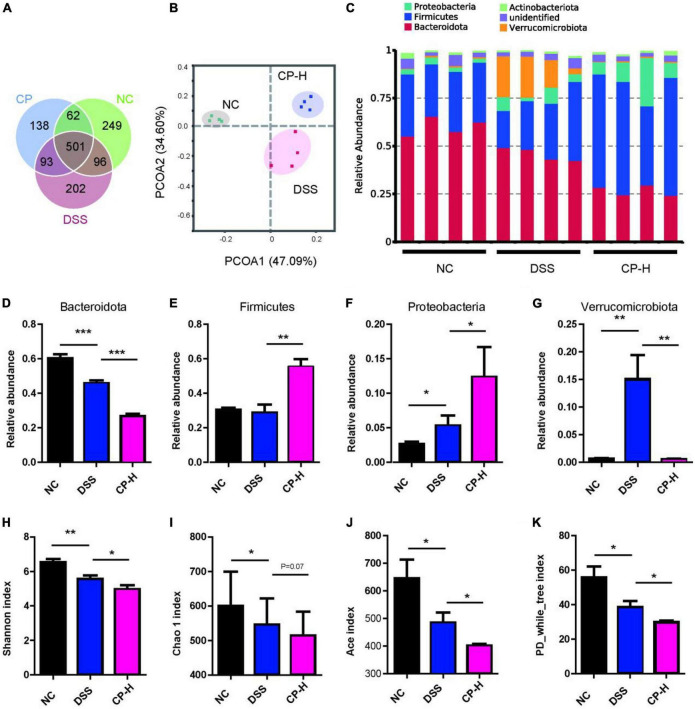
CP changed composition of gut microbiota. **(A)** Common OTUs for three groups. **(B)** PCoA analysis (*n* = 4). **(C)** Composition of microbiota at phylum level. Relative abundance of *Bacteroidota* **(D)**, *Firmicutes* **(E)**, *Proteobacteria* **(F)** and *Verrucomicrobiota* **(G)**. Shannon index **(H)**, Chao 1 index **(I)**, Ace index **(J)** and PD_while_tree index **(K)** for three groups. Compare with DSS group, *P < 0.05, **P < 0.01, ***P < 0.001.

The gut microbiota in the intestinal cavity is in close contact with the colonic epithelial barrier and regulates the host immune response through its effect on the intestinal epithelial barrier (26–28). According to the results of the effect of CP on the intestinal barrier (Figure 2), the effect of CP on colitis may not be dose-dependent, suggesting that it should be explored in depth. Therefore, we used 16S rRNA sequence analysis to observe the effect of CP (high dose) on the gut microbiota composition of DSS-induced colitis mice. The common and unique OTUs are shown in Figure 3A. The CP administrated group has the most overlap of OTUs with DSS-induced group. The weighted UniFrac Principal coordinates (PCoA) analysis revealed a markedly distinct colon gut microbiota landscape in mice in the NC, DSS and CP groups (Figure 3B). At the phylum level, the microbiota composition of DSS treated mice changed significantly, while CP treatment further exacerbated this change to a certain extent (Figure 3C). Compared with mice in NC group, DSS treatment significantly reduced the level of *Bacteroidota* and increased the level of *Proteobacteria*, and CP administration further promoted this change (Figures 3D,F). At the same time, CP treatment increased the change of *Firmicutes*, even if DSS induction did not cause its change (Figure 3E). DSS induction caused the increase of *Verrucomicrobiota* and CP administration could reduce this increase (Figure 3G). The Shannon’s diversity index, Chao 1 index, Ace index and PD-While-Tree index which reflected the community richness were all decreased in CP-H group compared with DSS group (Figures 3H–K). All these results indicated that the gut microbiota structure in mice with colitis was significantly impacted by oral CP administration.

**FIGURE 4:**
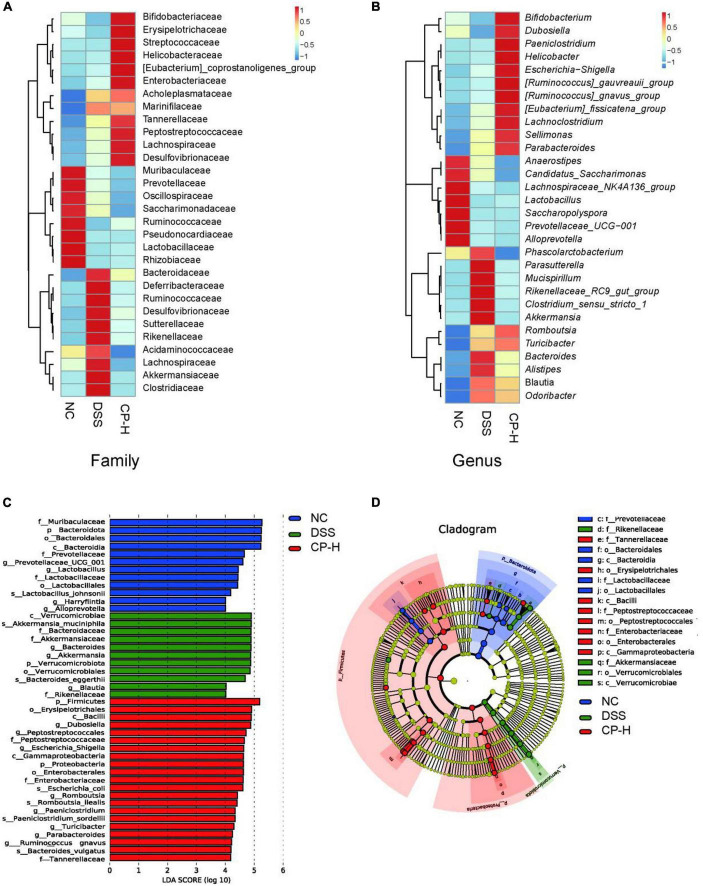
Change of gut microbiota by CP treatment. Heatmap of microbiome species abundance at the family **(A)** and genus level **(B)**. Color intensity was used to represent the abundance of bacteria. **(C)** Linear discriminant analysis (LDA) effect size (LEfSe) and **(D)** cladogram of species annotated by OTU were used to identify deferentially abundant taxa. Family (f), Genera (g), Class (c), Phylum (p), Order (o), Species (s) with *P* < 0.05 and LDA score (log 10) > 2 were considered significant.

The composition of gut microbiota for three groups at family and genus levels was further analyzed. At the family level, *Bifidobacteriaceae, Erysipelotrichaceae, Streptococcaceae, Helicobacteraceae, Coprostanoligenes, Enterobacteriaceae, Acholeplasmataceae, Marinifilaceae, Tannerellaceae, Peptostreptococcaceae, Lachnospiraceae* and *Desulfovibrionaceae* were obviously increased after CP treatment in mice with colitis (Figure 4A). Meanwhile more *Bifidobacterium*, *Dubosiella*, *Paeniclostridium*, *Helicobacter*, *Escherichia-Shigella*, *Gauvreauii*, *Gnavus*, *Fissicatena*, *Lachnoclostridium*, *Sellimonas* and *Parabacteroides* were appeared in CP-H group at the genus level (Figure 4B). Linear discriminant analysis (LDA) effect size (LEfSe) was used to identify the taxa, which could be significantly affected in the three groups at the family (f), genera (g), class (c), phylum (p), order (o) and species (s) levels (Figures 4C,D). We identified 44 bacterial taxa which were significantly different between the three groups. DSS group had a higher abundance of *Akkermansia* on a species level, as well as *Akkermansiaceae* on a family level. We therefore suggest that the *Akkermansia* bacterial may serve as potential microbiota markers for DSS group. Conversely, CP-H group revealed a larger abundance of *Peptosreptococcaceae* and *Enterobacteriaceae* on a family level. These results further indicated that CP administration altered the relative abundance of gut microbiota and overall components.

### Collagen peptide increases the expression of proinflammatory factors in DSS-induced colitis mice

**FIGURE 5:**
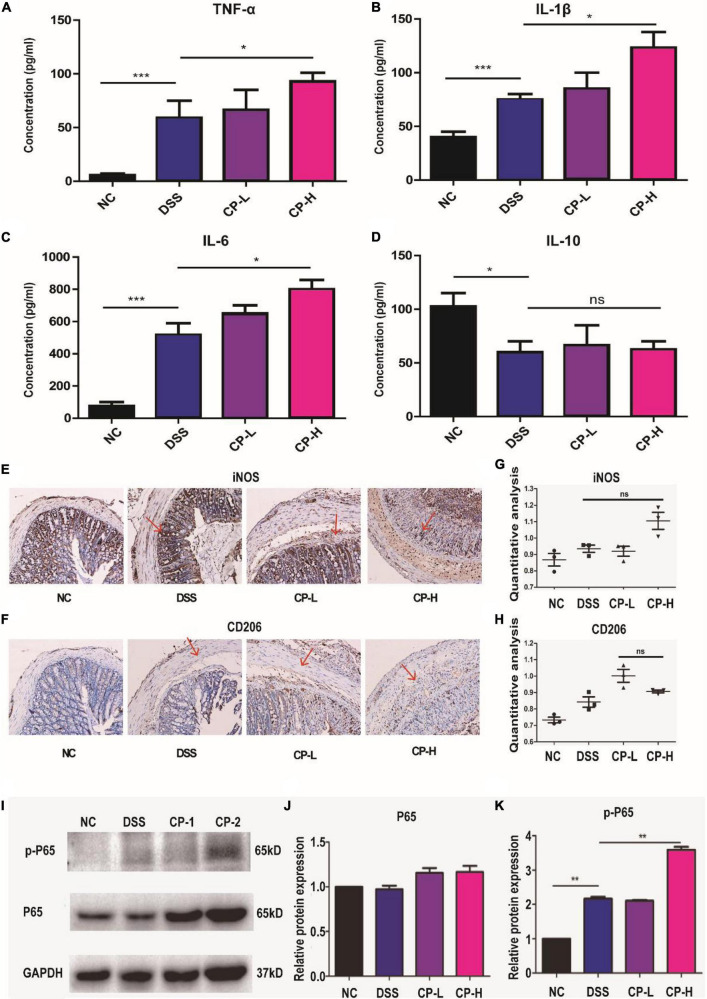
CP enhanced the inflammatory response in DSS-induced colitis. The expression of level of TNF-α **(A)**, IL-1β **(B)**, IL-6 **(C)** and IL-10 **(D)** in serum measured by ELISA (*n* = 5). Representative immunohistochemistry staining images for CD206 **(E)** and iNOS **(F)**. Panels **(G,H)** are quantitative analysis of panels **(E,F)** by IntDen/Area using Image J software. Western blot results of p-P65, P65 **(I)** and the quantitative analysis of western blot image for P65 **(J)** and p-P65 **(K)** in colonic tissue samples. Compare with DSS group, *P < 0.05, **P < 0.01, ***P < 0.001, ns: no significant.

To further assess the impact of CP on systemic and intestinal inflammatory response, pro-inflammatory and anti- inflammatory cytokines in the plasma were measured. The concentrations of TNF-α (Figure 5A), IL-1β (Figure 5B), IL-6 (Figure 5C) were significantly increased in CP-H group than DSS group. However, there was no difference in the concentrations of IL-10 in all three DSS groups (Figure 5D). Since innate immune response plays important roles in infection and inflammation and macrophages are one crucial member of host innate defense system, the infiltration and polarization of macrophages in intestine were detected (Figures 5E,F). Immunohistochemical staining results indicated more iNOS positive M1 macrophages were present in intestine submucosa layer of CP-H group compared with DSS group (Figures 5E,G). Meanwhile a slight increase of CD206 positive M2 macrophages was shown in CP-L group comparing with DSS group (Figures 5F,H). Inflammation related NF-κB signaling was also found to be further activated in intestine tissues of CP-H group as more p-P65 were shown in CP-H group compared with DSS group (Figures 5I–K). Collectively, these results indicated that the expression of proinflammatory factors were increased by CP in DSS-induced colitis mice and closely related to macrophages polarization.

### Collagen peptide aggravate inflammation in macrophages through NF-κB signaling

**FIGURE 6:**
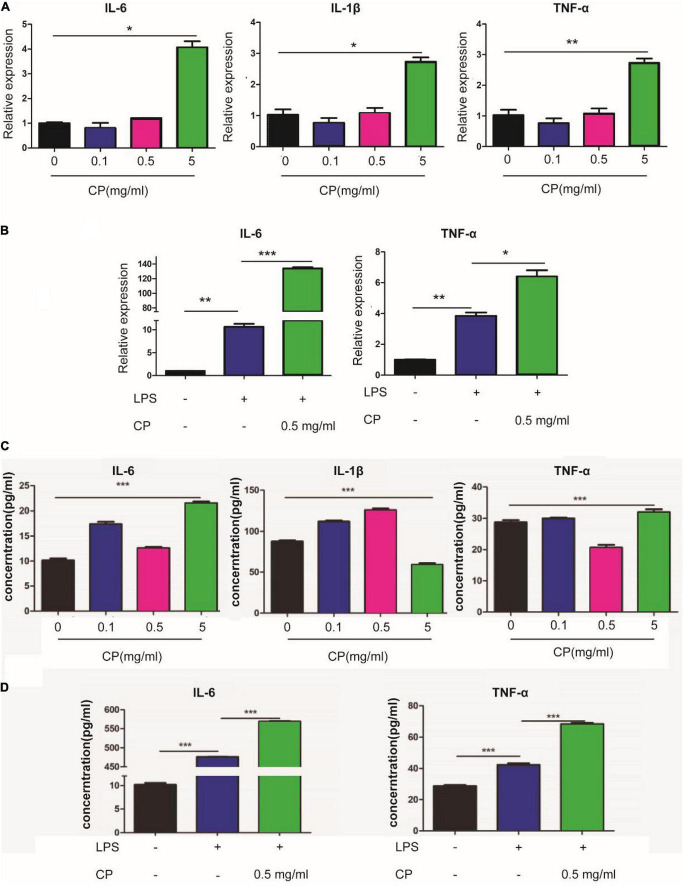
CP aggravate inflammation in macrophages using Raw264.7 cells. The mRNA expression changes of IL-6, IL-1β and TNF-α after 24 h treatment with 0.1, 0.5 or 5 mg/ml CP in murine Raw264.7 cells **(A)** or LPS-stimulated Raw264.7 cells **(B)**. The ELISA assay for expression changes of IL-6, IL-1β and TNF-α after 24 h treatment with 0.1, 0.5 or 5 mg/ml CP in murine Raw264.7 cells **(C)** or LPS-stimulated Raw264.7 cells **(D)**. *P < 0.05, **P < 0.01, ***P < 0.001.

**FIGURE 7:**
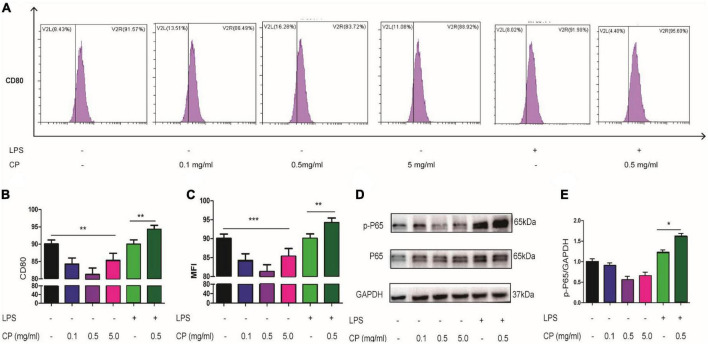
CP aggravate inflammation in macrophages through NF-κB signaling. Representative flow cytometry analysis of CD80 levels in different treatment groups of Raw264.7 cells **(A)**. The proportion of CD80-positive cells of three independent experiments **(B)**. The phagocytosis changes of Raw264.7 cells with or without CP treatment indicated by relative MFI (mean fluorescence intensity) from FACS **(C)**. Western blot results of p-P65, P65 **(D)** and the quantitative analysis of western blot image for p-P65, P65 **(E)** in Raw264.7 cells. *P < 0.05, **P < 0.01, ***P < 0.001.

To reveal the roles of collagen peptide on macrophages *in vitro*, the different concentrations of CP was added to the culture medium of unstimulated murine macrophage cell line Raw264.7. Real-time PCR showed that low and medium concentrations of CP had nearly no impact on the expression of pro-inflammatory genes such as IL-6, IL-1β and TNF-α while high concentration of CP slightly increased the expression of these pro-inflammatory genes in unstimulated murine macrophages (Figure 6A). Next, we added medium concentration of CP to LPS-stimulated macrophages which was a typical M1 macrophage. We found that CP could enhance the expression of IL-6 and TNF-α with LPS synergistically (Figure 6B). The expression trend of pro-inflammatory cytokines (IL-6, IL-1β and TNF-α) obtained by ELISA analysis was consistent with that of Real-time PCR (Figures 6C,D). Flow cytometric analysis also revealed that the number of CD80-positive cells was increased after CP treatment in M1 macrophages (Figures 7A,B). Also, the phagocytosis function of M1 macrophages was tested after CP addition. As the mean fluorescence intensity shown, more phagocytosis took place in CP treated M1 macrophages (Figure 7C). Next, we checked the classical inflammation related NF-κB signaling changes in M1 macrophages. CP treatment remarkably increased the p-P65 in M1 macrophages compared to unstimulated macrophages using murine Raw264.7 cells (Figures 7D,E). In conclusion, CP addition to stimulated macrophages could aggravate inflammation by increasing the number of CD80-positive macrophages and enhance the expression of pro-inflammatory genes by NF-κB signaling.

## Discussion

UC is a disease related to immune response and the destruction of gut microbiota will affect the immune system (29, 30). DSS is a chemical colitogen with anticoagulant properties, it is the most widely used chemical for colitis mouse model due to its rapidity, simplicity, reproducibility, and controllability (31). The mechanism by which DSS induces colitis is the result of damage to the epithelial monolayer lining the large intestine allowing the dissemination of proinflammatory intestinal contents (e.g., bacteria and their products) into underlying tissue (32). Previous studies have shown that CP, as a safe food additive, has multiple biological activities in animal models and healthy people (33). However, whether the effect of CP on inflammatory UC is positive or negative needs to be studied. Herein, DSS-induced colitis symptoms were significantly accelerated after treatment with different concentration of CP (Figure 1). This indicated that taking marine-derived collagen related health food and nutrients (such as CP) in the state of excessive inflammatory response under colonic mucosa is conducive to the progress of colitis.

A variety of cytokines participate in the coordination of the immune system and respond to endogenous or exogenous stimuli (34–36). Previous studies have showed that CP derived from enzymatic hydrolysate of Salmo salar skin had anti-inflammatory effects in LPS-induced Raw264.7 cell model (37). In addition, sea cucumber peptides inhibited NF-κB/MAPK signaling and inducing HO-1 in Raw264.7 macrophages to exert anti-inflammatory activity was reported (38). However, in the present study, we found that the expression of inflammatory cytokines was increased in the DSS group, while the treatment of CP further increased the inflammation induced by DSS (Figures 5A–C), indicating that CP further stimulated the inflammatory response under high inflammatory response. The results of cell model were consistent with DSS induced colitis model. If macrophages were pre-incubated with collagen peptides and then stimulated with LPS, they were tent to decrease inflammation. However, the inflammation was aggravated if LPS stimulation preceded CP incubation (Figure 6).

The change of gut microbiota in patients with colitis is closely related to infection. Numerous studies have shown that colitis may be affected by changes in microbial communities caused by diet or other factors (6, 39, 40). In this study, we found that DSS or CP treatment had a significant effect in colonic microbial composition. It should be noted that in the DSS group and CP treatment group, the pro-inflammatory bacteria (*Proteobacteria* and *bacteroidota* at the phylum level, *Helicobacter* and A*listipes* at the genus level) were increased significantly, and the anti-inflammatory bacteria (*Lactobacillus* in genus level) were decreased significantly (Figures 3C,D, 4B). In addition, CP can significantly improve the bacterial level of *Bifidobacterium* in genus level. We speculated that in the healthy state of the host, the increase of *Bifidobacterium* caused by CP administration is dominant, thus playing an anti-inflammatory role. In the inflammatory state of the body, the change of pro-inflammatory bacteria plays a leading role, offsetting the anti-inflammatory effect of CP and showing the promotion of inflammation.

The intestinal barrier function is realized by the TJ of epithelial cells and mucus layer (41, 42). The mucous layer can keep intestinal bacteria away from epithelial cells, and the TJ of epithelial cells can keep exogenous stimulation into the lamina propria, causing the body’s inflammatory response (43, 44). In this study, we observed that the expression of MUC2 and TJ protein decreased and the mucus layer became thinner in the DSS group (Figure 2). After DSS treatment, the number of mucolytic bacteria such as *Akkermansia* was increased, which may also lead to the thinning of mucus layer. However, the relative abundance of *Akkermansia* was not further increase in CP treatment group (Figure 4B). This may be due to the lack of mucin, which limits the growth of *Akkermansia* and its potential mechanism remains to be further studied.

**FIGURE 8:**
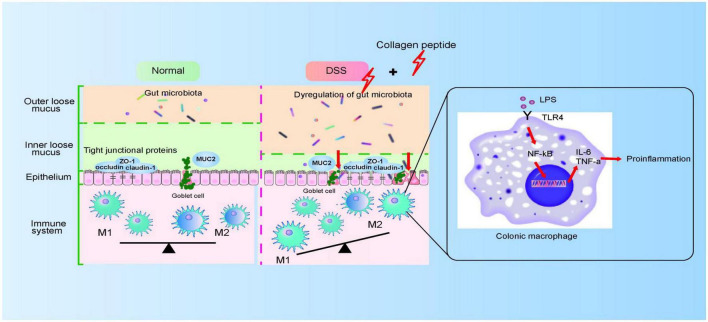
The proposed model for CP on colitis. CP disturbs gut microbiota and impairs colonic epithelial barrier, which then triggers the activation of macrophage and aggravated colitis progression.

Macrophages in resting state will undergo pro-inflammatory differentiation under the stimulated by LPS (45). LPS activates NF-κB signaling pathway by binding to TLR4 receptor and activates macrophages, thus releasing several cytokines (such as TNF-α, IL-6 and IL-1β). The released proinflammatory cytokines further increase the permeability of epithelial cells and further enhance intestinal mucosal injury (46, 47). In this study, we observed that CP treatment further promoted the activation of macrophages in the DSS-induced colitis mice (Figure 5). Because the intestinal destruction is too severe in DSS-induced colitis, it is difficult to study the mechanism using lymph node inflammatory cells *in situ*. Therefore, in this study, Raw264.7 cell model was used to prove that CP activates NF-κB signaling pathway and aggravates the inflammatory response of macrophages (Figure 6). Therefore, we proposed that CP treatment can change the composition of gut microbiota, maintain the destruction of intestinal barrier function induced by DSS (such as decrease expression of TJ proteins and MUC2). The disrupted epithelial barrier allows proinflammatory bacteria to pass through and cause LPS/TLR4/NF-κB signaling pathway mediated macrophage activation, thereby promoting the progression of colitis (Figure 8).

## Conclusion

In conclusion, our results showed that CP treatment in DSS-induced colitis can maintain the destruction of intestinal barrier function, change the composition of gut microbiota, trigger immune system response, and then promote the progress of colitis. This study had increased our understanding of the interaction between microbiota and host, expanded the regulatory role of the microbiota in inflammatory bowel disease. This suggested that we should avoid eating collagen related products from marine sources when treating colitis, which provides a new strategy for the adjuvant treatment of colitis.

## Data availability statement

The original contributions presented in the study are publicly available. This data can be found here: (https://www.ncbi.nlm.nih.gov/) PRJNA844568.

## Ethics statement

This animal study was reviewed and approved by Qingdao University.

## Author contributions

SL and NH designed the study. XL, LC, and SY performed the experiments related to mice model. XL, GF, and GS performed the experiments related to cell model. NH and XL wrote the manuscript. SL, NH, XL, LC, and SY reviewed and edited the manuscript. All authors approved the final manuscript.
