# Supplementary material for: Collagen peptide promotes DSS-induced colitis by disturbing gut microbiota and regulation of macrophage polarization
Source: Front Nutr. 2022 Oct 13;9:957391. doi: 10.3389/fnut.2022.957391 (PMC9608506; doi:10.3389/fnut.2022.957391)
Supplement: Supplementary file 1 [file Table_1.DOCX]

**Table. S1 List of primers sequences.**

| **Gene name** | **Primers sequence** |
| --- | --- |
| GAPDH | F: GGTTGTCTCCTGCGACTTCA |
|  | R: TGGTCCAGGGTTTCTTACTCC |
| MUC2 | F: GACGGCGATGTCTACCGATT |
|  | R: GGTCAGCAGCCTCTCACATT |
| IL-6 | F: TAGTCCTTCCTACCCCAATTTCC |
|  | R: TTGGTCCTTAGCCACTCCTTC |
| IL-1β | F: AGCTACGAATCTCCGACCAC |
|  | R: CGTTATCCCATGTGTCGAAGAA |
| TNF-α | F: CCTCTCTCTAATCAGCCCTCTG |
|  | R: GAGGACCTGGGAGTAGATGAG |
|  |  |

F: Forward; R: Reverse
